# Current status and technological prospect of photodetectors based on two-dimensional materials

**DOI:** 10.1038/s41467-018-07643-7

**Published:** 2018-12-10

**Authors:** Gerasimos Konstantatos

**Affiliations:** 1 ICFO—Institut de Ciencies Fotoniques, The Barcelona Institute of Science and Technology, Av. Carl Friedrich Gauss, 3, 08860 Castelldefels (Barcelona), Spain; 2 ICREA—Institució Catalana de Recerca i Estudis Avançats, Passeig Lluís Companys 23, 08010 Barcelona, Spain

## Introduction

Photodetectors are an essential component in a vast number of devices and applications nowadays for they are the cornerstone component of image sensors, ambient sensors in consumer electronics, in spectrometry, biomedical imaging, food and manufacturing process monitoring, just to name a few. The biggest portion of the photodetector market is currently served by silicon (CMOS-based) photodetectors in view of their high performance, low cost, maturity and high level of integration with electronics. Applications that require photodetection in the infrared, i.e., beyond silicon’s bandgap, are currently relying on exotic semiconductors such as III-V InGaAs or HgCdTe systems that can cover a spectral beyond 10 μm. Such technologies, albeit offering high performance detectors, suffer from severe manufacturing costs and integration issues, and therefore are limited only to niche low-volume, high-value markets. This has a major impact on several applications (night vision, infrared spectrometry, infrared imaging) that would provide indispensable information to consumers and automotive industry in terms of health, safety and security. This grand challenge is one that two-dimensional (2D) materials are called for to address, and offer an opportunity to bring about a significant market value growth as well as profound socioeconomic impact. Among the various optoelectronic applications considered for 2D materials, one that has therefore attracted significant attention is that of photodetectors^1^. Graphene, an atomically thin semimetallic material with extraordinarily large carrier mobility, flat broadband absorption, and electrostatically tunable carrier concentration, offers unique properties that can be leveraged for photodetection. 2D semiconductor analogs also provide very high carrier mobilities—especially taking into account their nearly atomic thin profiles—and a spectral coverage both in the visible and in the infrared wavelength range, depending upon the material selection. A common additional feature of those materials is their ‘form factor’ and the resulting possibility to enable high performance optoelectronic devices that are ultra-low-weight, flexible, and therefore suited for seamless integration in a variety of substrates, either rigid or flexible, single crystalline or amorphous etc.

## 2D photodectors: prospects and challenges

Intensive efforts have been underway, and should remain in full speed, towards photodetectors with unique features over traditional technologies. One way for 2D materials to prove their added value in the photodetector technology landscape is to provide devices with a form factor that cannot be implemented with standard single crystalline technologies, or devices whose performance is cost- and performance-competitive over current technologies. The latter can be distinguished in two main features: either achievement of very high modulation frequencies (relevant for data communication applications), or of very high sensitivity (relevant for imaging, remote sensing and spectrometry applications). The former has already been demonstrated in several cases based on graphene photodetectors^2–5^ and 2D/transition metal dichalcogenides (TMD) heterojunction photodetectors^6^ reporting modulation bandwidths in excess of 60 GHz^7^. The extremely high carrier mobility and carrier relaxation in graphene has been exploited in this case, providing a valid example of matching a unique material property with a key performance indicator of the detector. Alternatively, high sensitivity photodetectors have been reported based on sensitizing graphene^8^ and/or 2D materials^9,10^ with other material platforms whose spectral coverage is dictated by that of the sensitizer material. In this case the atomic thin profile of the 2D material that constitutes the transistor channel with very high mobility and gate-tunable Fermi level has been instrumental in realizing photodetectors with very high gain and sensitivity comparable to that of single crystalline counterparts.

**Fig. 1:**
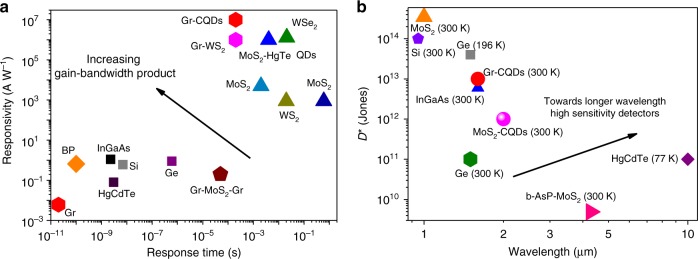
Overview of performance metrics of standard and 2D-based photodetector technologies. **a** Various photodetector technologies plotted against their corresponding responsivity and time response. The top left region corresponds to high gain-bandwidth-product detectors and should be the ultimate performance target of future 2D-based photodetectors. **b** Plot of the specific detectivity D* of various photodetector technologies versus their absorption onset (bandgap). 2D material-based photodetectors have the potential to address the grand challenge in photodetection towards low-cost, high sensitivity infrared detectors

There has been a significant body of published literature on 2D material-based photodetectors, a phenomenon quite typical when a new material platform is discovered. Yet, in the process of maturing, there has to be a progressive focus towards solutions that are uniquely enabled by this innovative platform, instead of competing with existing technologies. When it comes to photodetectors there is a set of key performance indicators that characterize the potential of a new technology to become disruptive. One important figure of merit is the sensitivity of the detector, in which three metrics have to be carefully measured under the same experimental conditions, and reported as a function of electrical bandwidth, temperature, and optical wavelength. These are the quantum efficiency, the responsivity and the noise current of the detector. The quantum efficiency is essential in that yields the number of primary electrical carriers that are generated per single incident photon and it is typically limited to unity (100%), unless multicarrier generation or avalanche effects take place. The responsivity is the electric current that flows per watt of incident optical power, and can reach very high values in the presence of a photoconductive gain mechanism. Yet, what finally determines the sensitivity of a detector is the combination of its responsivity and noise current. It is noteworthy that in a significant portion of published literature the latter is calculated at the shot noise limit, a condition that is rarely met in photodetectors, in particular photoconductive detectors. In addition to this, the presence of 1/*f* or flicker noise in graphene and 2D materials is now well documented and represents the most dominant noise source in such devices. Thus, it is crucial that published literature report sensitivity performance (typically expressed as specific detectivity, or D*) based on experimental noise measurements, particularly when claiming record performance devices. A large body of literature has reported the achievement of ultrahigh gain and consequently record high responsivity values^9,12,13^. Yet the cost that needs to be paid for reaching a high gain is often underestimated: that of the photodetector speed, which is of paramount importance for a large number of applications. For example, in imaging technologies, at least video frame rates ~30–60 Hz are required to enable taking high quality video without ghosting effects, whereas even higher frame rates on the order of 10–100 KHz are required for machine night vision applications. In most of the cases the detector itself should be even faster than that in order to allow for real-time read-out and processing in image sensor arrays. Gain is proportional to the mobility of the transistor channel and the carrier lifetime, the latter determining the speed of the detector. The origin of very high gain is often due to the very long lifetimes, in some cases on the order of seconds or tens of seconds. Such long lifetimes will likely prevent the photodetector from addressing a real world application, thus efforts towards gain improvement should be focused on increasing carrier mobility rather than prolonging carrier lifetime. Thus, the ultimate figure of merit to be looked upon is the gain bandwidth product, and not the gain itself. Figure 1a shows the responsivity and time response of various photodetector technologies (including 2D-based as well as currently employed technologies) and illustrates the path that needs to be followed for competitive emerging technologies. Likewise, in the case of photodiodes or graphene-based *p*-*n* junction detectors, the speed of a photodetector is of paramount importance especially in time-of-flight remote sensing or data communications. Again, the reports on the speed of the detector have to be documented at relevant conditions, and followed by a full characterization of the detector in achieving high speed without sacrificing other key figures of merit such as the quantum efficiency and the sensitivity. A fast photodetector must also be sensitive enough and operate at realistically relevant conditions (in terms of temperature, form factor etc.). As mentioned earlier, 2D materials hold great promise to disrupt the infrared detector market exploiting graphene’s broadband absorption, or through the combination with the appropriate infrared sensitizers. Figure 1b illustrates the prospect of 2D-based photodetectors in overcoming, in performance and cost, the currently employed infrared semiconductor technologies based mainly on InGaAs and HgCdTe photodiodes. The possibility offered by the 2D materials to operate high sensitivity detectors not in a photodiode mode but in phototransistors may offer additional advantages in achieving compact highly sensitive infrared detectors and bolometers.

## 2D photodetectors: the path to the market

When it comes to disruptive technologies and their entry into existing markets or opening new markets, besides their competitive aspects in key performance indicators or cost, another equally, if not more important, aspect is their manufacturing readiness levels. This is particularly relevant in the case of 2D materials, in view of their unique form factor and requirements of not-standardized integration processes, especially in CMOS foundries. There are already a few strong examples of their potential to augment or even replace current photodetector technologies^6,11^. To this end, a consolidated and convincing roadmap is required to warrant the successful adoption of 2D material-based technologies in production lines. Significant efforts are still needed to address doping non-uniformities, batch-to-batch variability arising from growth or even transfer variability issues, as well as hysteretic effects that are sometimes present in graphene and 2D materials due to their interaction with their local environment. Solutions to those challenges should also be scalable, and should not introduce additional complexity in manufacturing. There is indeed a substantial potential for 2D materials to play a crucial role in the next generation of photodetectors, image sensors, optical transceivers, spectrometers etc. Whether this becomes a reality will depend largely on the successful rise of those initial demonstrators both in technology- and manufacturing- readiness levels through synergism of researchers with semiconductor foundries and key industrial players.
